# Diagnostic performance of [^68^Ga]Ga-FAPI-04 PET vs. [^18^F]FDG PET in detecting lymph node metastasis in digestive system cancers: a head-to-head comparative meta-analysis

**DOI:** 10.3389/fmed.2025.1541461

**Published:** 2025-03-21

**Authors:** Huo Li, Zhognzhuan Li, Jing Qin, Shijiang Huang, Shufen Qin, Zhixin Chen, Rong Ouyang

**Affiliations:** ^1^Gastroenterology, The Fourth Affiliated Hospital of Guangxi Medical University/Liuzhou Worker’s Hospital, Liuzhou, China; ^2^Department of General Medicine, Liuzhou People’s Hospital, Liuzhou, China

**Keywords:** digestive system cancers, [^68^Ga]Ga-FAPI-04 PET, [^18^F]FDG PET, lymph node metastasis, meta-analysis

## Abstract

**Purpose:**

This meta-analysis aimed to compare the diagnostic effectiveness of [^68^Ga]Ga-FAPI-04 PET and [^18^F]FDG PET for detecting lymph node metastasis in digestive system cancer patients.

**Methods:**

A comprehensive search of PubMed, Web of Science, and Embase databases was conducted to identify relevant articles up to June 2024. Studies were included if they evaluated the diagnostic performance of [^68^Ga]Ga-FAPI-04 PET and [^18^F]FDG PET in detecting lymph node metastasis in digestive system cancer patients. Sensitivity and specificity were assessed using the DerSimonian and Laird method and were transformed using the Freeman-Tukey double arcsine transformation.

**Results:**

Fifteen articles, encompassing a total of 617 patients, were included in this study. The overall sensitivity of [^68^Ga]Ga-FAPI-04 PET for diagnosing lymph node metastasis in digestive system cancers was 0.82 (95% CI: 0.67–0.93), and the specificity was 0.91 (95% CI: 0.84–0.97). In comparison, the sensitivity of [^18^F]FDG PET was 0.51 (95% CI: 0.38–0.63), with a specificity of 0.81 (95% CI: 0.64–0.94). These results suggest that [^68^Ga]Ga-FAPI-04 PET has a significantly higher sensitivity (*P* < 0.01) and similar specificity (*P* = 0.20) compared to [^18^F]FDG PET in detecting lymph node metastasis in digestive system cancers.

**Conclusion:**

Our meta-analysis indicates that [^68^Ga]Ga-FAPI-04 PET has higher sensitivity and similar specificity compared to [^18^F]FDG PET in diagnosing lymph node metastasis in digestive system cancers. However, the high heterogeneity among the studies may impact the robustness of the current evidence. Therefore, future research should prioritize larger prospective studies with more diverse populations and specific cancer subtypes to draw more definitive conclusions.

**Systematic review registration:**

https://www.crd.york.ac.uk/PROSPERO/view/CRD42024572412, Unique Identifier: CRD42024572412.

## 1 Introduction

Digestive system cancers are a significant health concern, impacting multiple organs and being widely prevalent (1). Gastrointestinal cancers account for over 26% of the global cancer incidence and are responsible for more than 35% of cancer-related deaths worldwide (2). In 2018, gastrointestinal tumors accounted for 4.8 million new cases and 3.4 million deaths, with Asia bearing 63% of cases and 65% of deaths. China alone contributed 38% of cases and 41% of deaths (2). Within China, four of the top five cancers leading to mortality are gastrointestinal, including liver cancer (12.85%), gastric cancer (12.48%), esophageal cancer (10.09%), and colorectal cancer (9.63%) (3). These statistics highlight the urgent need for early detection and accurate staging of gastrointestinal cancers(4). While histopathology is the gold standard, advancements in imaging technologies offer a promising non-invasive alternative (5).

Traditional imaging techniques include ultrasound, computed tomography (CT), and magnetic resonance imaging (MRI) (6). Due to their cost-effectiveness and accessibility in clinical applications, these methods are widely employed for the detection of gastrointestinal malignancies. However, they possess certain limitations. For instance, ultrasound may produce artifacts due to the intestines’ complex anatomy and high gas content, reducing accuracy (7, 8). Furthermore, current endoscopic ultrasound is limited by insufficient penetration depth and difficulty in observing distant lymph nodes, making accurate assessment challenging (9). Enhanced CT and MRI may fail to accurately differentiate small nodules from atypical lesions in patients with hepatocellular carcinoma (HCC) (10). Furthermore, these imaging techniques may not offer adequate functional data for precise lymph node assessment. These methods frequently exhibit limitations in detecting lymph node metastases and small lesions, highlighting the urgent need for the development of enhanced diagnostic tools (11).

Positron emission tomography (PET) has been a critical tool in molecular imaging over the past decade, frequently employed in the detection of gastrointestinal cancers (12, 13). The ^18^F-FDG tracer, which targets abnormal glucose metabolism in tumors, is a key tool in PET imaging. It is extensively used for diagnosing malignant tumors and evaluating treatment effectiveness (14). Recent studies have identified limitations of ^18^F-FDG tracers in diagnosing gastrointestinal cancers, particularly in distinguishing between inflammation and malignancy (14–16). Nonspecific lymph node uptake may cause false positives and incorrect treatment decisions (17). Recent studies show that high levels of fibroblast activation protein (FAP) in cancer-associated fibroblasts are linked to tumor growth, metastasis, and prognosis. As a result, FAPI has become a novel imaging agent and has been used in clinical practice since 2018 (18). Ga-labeled FAPI tracers (such as [^68^Ga]Ga FAPI-04 and [^68^Ga]Ga FAPI-46) have shown rapid tumor uptake, with [^68^Ga]Ga FAPI-04 being particularly notable (19). Its potential applications have made [^68^Ga]Ga FAPI-04 the focus of growing research interest. [^68^Ga]Ga FAPI-04 is being studied as a potential alternative to the well-established [^18^F]FDG in gastrointestinal oncologic PET imaging. Previous studies conducted by Ouyang et al. and Wang et al. indicated that [^68^Ga]Ga FAPI-04 PET exhibit higher sensitivity than [^18^F]FDG PET in diagnosing primary gastrointestinal tumors or gastric cancer (20, 21). However, its diagnostic performance for detecting lymph node metastasis in gastrointestinal tumors has not been reported. Lymph node metastasis is a well-established indicator of cancer spread, and accurate N staging of the tumor is essential for developing effective treatment strategies (22).

The relative sensitivity of [^68^Ga]Ga-FAPI-04 PET compared to [^18^F]FDG PET in diagnosing lymph node metastasis in digestive system cancers is currently under debate, given the conflicting findings in the literature. The study by Lin et al. indicates that the diagnostic performance of [^68^Ga]Ga FAPI-04 PET is comparable to [^18^F]FDG PET for lymph node metastasis in digestive system cancers (23); however, Gündoğan et al. found that [^68^Ga]Ga FAPI-04 PET has higher sensitivity than [^18^F]FDG PET (24). Therefore, we conducted a systematic review and meta-analysis, rigorously collecting and analyzing all head-to-head eligible studies to provide a more conclusive assessment.

## 2 Methods

This meta-analysis strictly followed the PRISMA-DTA guidelines for Diagnostic Test Accuracy (25). Additionally, the study protocol was registered with the PROSPERO network under the identifier CRD42024572412.

### 2.1 Search strategy

A systematic search was conducted across three major English electronic databases—PubMed, EMBASE, and Web of Science—from their inception through June 2024. The search strategy utilized specific terms including: (1) PET or positron emission tomography, (2) ^68^Ga-FAPI, FAPI-04, FAPI, fibroblast activation protein, or FAP, and (3) digestive, gastric, gastrointestinal, pancreatic, pancreas, colorectal, hepatic, hepatocellular, or liver. Detailed search terms are listed in Supplementary Table 1. In addition, the reference lists of the selected articles were manually reviewed to identify any additional relevant studies.

### 2.2 Inclusion and exclusion criteria

The selection criteria were defined according to PICOS: participants (P) were patients with digestive system tumors; the index test (I) was [^68^Ga]Ga-FAPI-04 PET; the comparator (C) was [^18^F]FDG PET; outcomes (O) were diagnostic accuracy with histopathology or follow-up imaging as reference standards; and study design (S) included both prospective and retrospective diagnostic studies published in English.

Studies were excluded based on the following criteria: (1) duplicate publications, (2) abstracts, editorial comments, letters, case reports, reviews, or meta-analyses, (3) irrelevant studies, and (4) studies lacking extractable data for true-positive (TP), false-positive (FP), true-negative (TN), and false-negative (FN) results. Additionally, studies using different radiotracers or PET without CT or MRI were excluded. In cases of potentially overlapping patient samples, the most recent publication was selected.

### 2.3 Quality assessment

According to the Quality Assessment of Diagnostic Accuracy Studies-2 (QUADAS-2) tool (26), two independent researchers evaluated the quality of the included studies. The QUADAS-2 tool assesses four critical domains: (1) patient selection, (2) index test, (3) reference standard, and (4) flow and timing. The risk of bias for each domain was categorized as “high risk,” “low risk,” or “unclear risk.”

### 2.4 Data extraction

The extracted data from the selected studies included several key elements: author, publication year, study location, type of radiotracer ([^68^Ga]Ga-FAPI-04 or [^18^F]FDG), study design (prospective or retrospective), analysis type (patient-based or lesion-based), reference standard (pathology or imaging follow-up), patient demographics (mean or median age, number of patients), cancer type, interval between radiotracer tests (median and range), and diagnostic outcomes such as true-positive (TP), false-positive (FP), true-negative (TN), and false-negative (FN) results.

Two reviewers independently extracted data from each study. Discrepancies were resolved through discussion and consultation with an experienced third reviewer, ensuring consensus and accuracy in the data extraction process.

### 2.5 Statistical analysis

Specificity and sensitivity were evaluated using the DerSimonian and Laird method, followed by normalization of the data using the Freeman-Tukey double arcsine transformation. This transformation helps to make the data more suitable for analysis by converting proportions (which can range from 0 to 1) into values that are more evenly distributed, thereby resembling a normal distribution (27, 28). The confidence interval was calculated using the Jackson method. Heterogeneity, both within and between groups, was assessed using the Cochrane Q test and I^2^ statistics. Significant heterogeneity was defined as (*P* < 0.10 or I^2^ > 50%) (29). When significant heterogeneity was detected, leave-one-out sensitivity analysis was conducted by sequentially excluding individual studies and reassessing specificity or sensitivity. Additionally, meta-regression analysis was performed to identify potential sources of this heterogeneity (30).

Funnel plots and the Egger test were employed to evaluate publication bias. Statistical significance was set at *P* < 0.05 for all analyses, except for the heterogeneity test, where a threshold of *P* < 0.10 was applied. All statistical analyses and figures were produced using R software (version 4.4.1).

## 3 Results

### 3.1 Study selection

**FIGURE 1:**
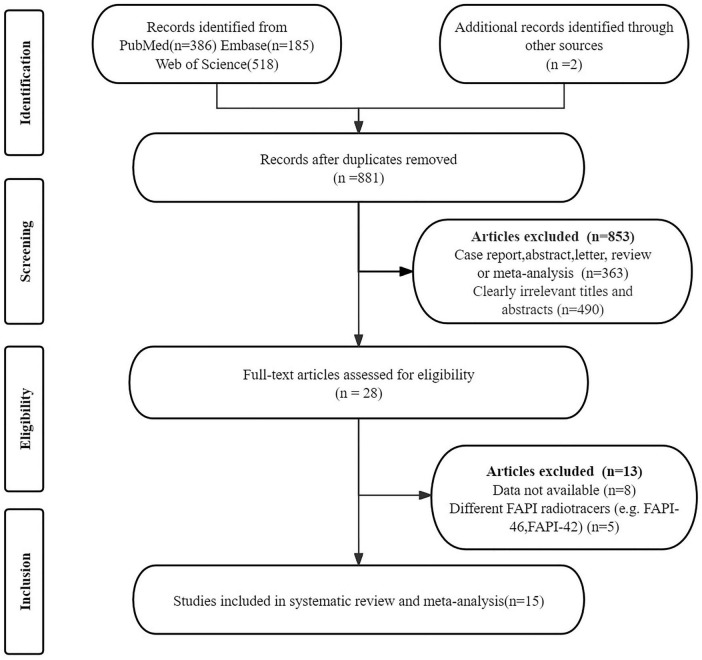
PRISMA flow diagram for literature search and study selection.

The initial search identified 1, 091 articles. After removing 210 duplicates and excluding 853 that did not meet the inclusion criteria, 28 articles remained for further consideration. Following a thorough review of the full texts, 13 articles were excluded for the following reasons: data (TP, TN, FP, and FN) not available (*n* = 8); articles using different FAPI radiotracers (e.g., FAPI-46, FAPI-42) (*n* = 5). The final analysis included 15 articles that evaluated the diagnostic performance of [^68^Ga]Ga-FAPI-04 PET and [^18^F]FDG PET (21, 23, 24, 31–42). Details of the article selection process are provided in Figure 1, in accordance with the PRISMA flow diagram.

### 3.2 Study description and quality assessment

This analysis included 15 studies that met the eligibility criteria. These studies collectively involved 617 patients diagnosed with digestive system cancers (ranging from 19 to 62, with a median of 40 years). The studies were published between 2021 and 2024. Among these, the study populations for 13(86.67%) of the articles were Chinese, while 2(13.33%) articles focused on populations from other countries, specifically India and Turkey. 13(86.67%) utilized PET CT scanners, while 2(13.33%) employed PET MRI scanners. 9 (60%) were retrospective studies, and 6 (40%) were prospective. Regarding analysis methods, 7 (46.67%) studies conducted patient-based analysis, 8(53.33%) employed lesion-based analysis. The reference standard was pathology and imaging follow-up in 5 (33.33%) articles, pathology in 10 (66.67%) articles. Table 1 summarizes the basic information and patient characteristics of the included studies, and Table 2 summarizes the technical aspects of included studies.

**TABLE 1:** The basic study characteristics and the patient characteristics from the included studies.

References	Intervention	Country	Study design	Reference standard	Analysis	No. of patients	Mean/median age	Cancer type	Interval day for both radiotracers median (range)
Pang et al. (39)	[^68^Ga]Ga-FAPI-04 PET/CT vs. [^18^F]FDG PET/CT	China	Retro	Pathology	LB	35	Mean ± SD: 56 ± 12	Gastric, duodenal, and colorectal cancer	2(1–6)
Gündoğan et al. (24)	[^68^Ga]Ga-FAPI-04 PET/CT vs. [^18^F]FDG PET/CT	Turkey	Pro	Pathology	PB	21	Median (range):61(40–81)	Gastric caner	<7
Lin et al. (23)	[^68^Ga]Ga-FAPI-04 PET/CT vs. [^18^F]FDG PET/CT	China	Pro	Pathology	LB	56	Mean ± SD: 63.8 ± 14.9	Gastric cancer	<7
Zhang et al. (42)	[^68^Ga]Ga-FAPI-04 PET/CT vs. [^18^F]FDG PET/CT	China	Retro	Pathology	LB	19	Mean ± SD: 56 ± 12(35–79)	Gastric cancer	NA
Pang et al. (40)	[^68^Ga]Ga-FAPI-04 PET/CT vs. [^18^F]FDG PET/CT	China	Retro	Pathology or follow-up imaging	LB	36	Median (range):60(48–71)	Pancreatic cancer	NA (1–6)
Li et al. (36)	[^68^Ga]Ga-FAPI-04 PET/CT vs. [^18^F]FDG PET/CT	China	Pro	Pathology or follow-up imaging	LB	47	Mean ± SD: 50.09 ± 10.98(33–80)	Biliary tract carcinoma	NA
Ding et al. (32)	[^68^Ga]Ga-FAPI-04 PET/CT vs. [^18^F]FDG PET/CT	China	Pro	Pathology	PB	49	Mean ± SD: 60.9 ± 8.9(NA)	Pancreatic ductal adenocarcinoma	NA
Du et al. (33)	[^68^Ga]Ga-FAPI-04 PET/MRI vs. [^18^F]FDG PET/MRI	China	Pro	Pathology	PB	40	Median (range):68(18–80)	Gastric cancer	2
Chen et al. (31)	[^68^Ga]Ga-FAPI-04 PET/CT vs. [^18^F]FDG PET/CT	China	Retro	Pathology and follow-up imaging	LB	34	Median (range):51(25–85)	Gastric signet-ring-cell carcinoma	2(1–7)
Liu et al. (37)	[^68^Ga]Ga-FAPI-04 PET/CT vs. [^18^F]FDG PET/CT	China	Retro	Pathology and follow-up imaging	LB	41	Median (range):51(19–75)	Gastric, duodenal, and colorectal cancers	<7
Wang et al. (21)	[^68^Ga]Ga-FAPI-04 PET/CT vs. [^18^F]FDG PET/CT	China	Retro	Pathology	LB	59	Median (range):59(29–77)	Gastric and colorectal cancer	<3
Prashanth et al. (41)	[^68^Ga]Ga-FAPI-04 PET/CT vs. [^18^F]FDG PET/CT	India	Retro	Pathology	PB	29	NA	Colorectal cancer	1 (1–3)
Li et al. (36)	[^68^Ga]Ga-FAPI-04 PET/CT vs. [^18^F]FDG PET/CT	China	Retro	Pathology and follow-up imaging	PB	51	Median (range):57(48–66)	Gastric, colon, rectal and appendiceal cancers	1
Jiang et al. (34)	[^68^Ga]Ga-FAPI-04 PET/CT or PET/MRI vs. [^18^F]FDG PET/CT or PET/MRI	China	Pro	Pathology	PB	38	Mean ± SD: 63.7 ± 15.3(25–86)	Gastric cancer	2
Miao et al. (38)	[^68^Ga]Ga-FAPI-04 PET/CT vs. [^18^F]FDG PET/CT	China	Pro	Pathology	PB	62	Median (range):64(24–75)	Gastric cancer	<9

PB patient-based; LB lesion-based; Pro prospective; Retro retrospective; NA not available.

**TABLE 2:** Technical aspects of included studies.

References	Types of imaging tests	Scanner modality	Ligand dose		TP, FP, FN, TN for [^68^Ga]Ga-FAPI-04 PET	TP, FP, FN, TN for [^18^F]FDG PET
			**[^18^F]FDG**	**[^68^Ga]Ga-FAPI-04**		
Pang et al. (39)	PET/CT	Discovery MI; GE Healthcare, Milwaukee, WI, United States	3.7MBq/Kg	1.8–2.2 MBq/Kg	TP:22, FP:10, FN:6, TN:46	TP:15, FP:6, FN:13, TN:50
Gündoğan et al. (24)	PET/CT	Discovery MI; GE Healthcare, Milwaukee, WI, United States	3.5–5.5 MBq/Kg	2.0 MBq/Kg	TP:21, FP:NA, FN:0, TN:NA	TP:15, FP:NA, FN:6, TN:NA
Lin et al. (23)	PET/CT	Biograph mCT64, Siemens Healthcare	3.7 MBq/Kg	111–185 MBq	TP:20, FP:0, FN:84, TN:512	TP:16, FP:12, FN:88, TN:509
Zhang et al. (42)	PET/CT	uMI780, United Imaging Healthcare	3.7 MBq/Kg	1.85 MBq/Kg	TP:75, FP:2, FN:0, TN:8	TP:32, FP:4, FN:33, TN:6
Pang et al. (40)	PET/CT	Discovery MI; GE Healthcare, Milwaukee, WI, United States	3.7 MBq/Kg	1.8–2.2 MBq/Kg	TP:18, FP:21, FN:4, TN:126	TP:13, FP:28, FN:9, TN:119
Lin et al.	PET/CT	NA	3.7 ± 0.19 MBq/Kg	2.04 ± 0.22MBq/Kg	TP:181, FP:12, FN:20, TN:43	TP:175, FP:10, FN:26, TN:33
Ding et al. (32)	PET/CT	NA	NA	NA	TP:9, FP:0, FN:7, TN:14	TP:5, FP:0, FN:11, TN:14
Du et al. (33)	PET/MRI	NA	5.5 MBq/Kg	5.5 MBq/Kg	TP:7, FP:5, FN:5, TN:12	TP:4, FP:3, FN:8, TN:14
Chen et al. (31)	NA	NA	281.2(203.5–358.9) MBq	194.3(133.2–281.2) MBq	TP:59, FP:11, FN:18, TN:405	TP:18, FP:10, FN:59, TN:406
Liu et al. (37)	PET/CT	NA	3.7 MBq/Kg	1.85 MBq/Kg	TP:92, FP:2, FN:0, TN:9	TP:31, FP:11, FN:61, TN:0
Wang et al. (21)	PET/CT	PHILIPS Vereos128, Philips Medical Systems, Inc., PET/CT)	3.7 MBq/Kg	1.5–1.8 MBq/Kg	TP:11, FP:2, FN:3, TN:48	TP:10, FP:15, FN:4, TN:64
Prashanth et al. (41)	PET/CT	Discovery MI; GE Healthcare, Milwaukee, WI, United States	3.7 MBq/Kg	1.8–2.2 MBq/Kg	TP:20, FP:NA, FN:0, TN:NA	TP:16, FP:NA, FN:4, TN:NA
Li et al. (36)	PET/CT	Biograph mCT; Siemens Healthineers)	3.7–5.5 MBq/Kg	1.85–3.7 MBq/Kg	TP:9, FP:NA, FN:2, TN:NA	TP:6, FP:NA, FN:5, TN:NA
Jiang et al. (34)	PET/CT or PET/MRI	PET/MR (uPMR790 TOF, United Imaging, China) or PET/CT (Biograph mCT, Siemens Healthineers, Germany; Ingenuity TF, Philips Healthcare, United States; uMI510, United Imaging, China	NA	111–185 MBq	TP:6, FP:1, FN:4, TN:13	TP:5, FP:1, FN:5, TN:13
Miao et al. (38)	PET/CT	Biograph Vision 450, Siemens Healthineers	3.7–4.44 MBq/Kg	1.85–2.96 MBq/Kg	TP:7, FP:1, FN:4, TN:8	TP:6, FP:2, FN:5, TN:7

TP, true positive; TN, true negative; FP, false positive; FN, false positive; NA, not available.

**FIGURE 2:**
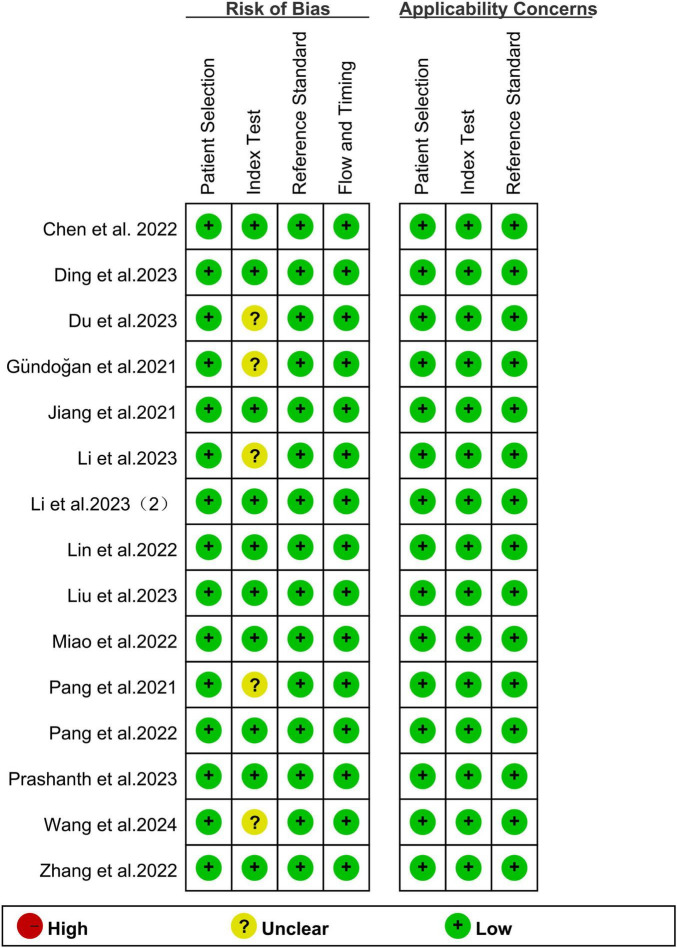
Summary of risk of bias and applicability concerns of all included studies according to QUADAS-2 tool.

The risk of bias for all studies was evaluated using the QUADAS-2 tool, as illustrated in Figure 2. Regarding the index test, 5 studies (33.33%) were classified as “unclear” due to insufficient information on the use of predefined thresholds. For other aspects, including patient selection, reference standard, flow and timing, and applicability concerns related to patient selection, the index test, and the reference standard, all studies were rated as “low” risk. Overall, no significant quality issues were identified in the included studies.

### 3.3 Comparing the sensitivity of [^68^Ga]Ga-FAPI-04 PET and [^18^F]FDG PET in detecting lymph node metastasis of digestive system cancers

**FIGURE 3:**
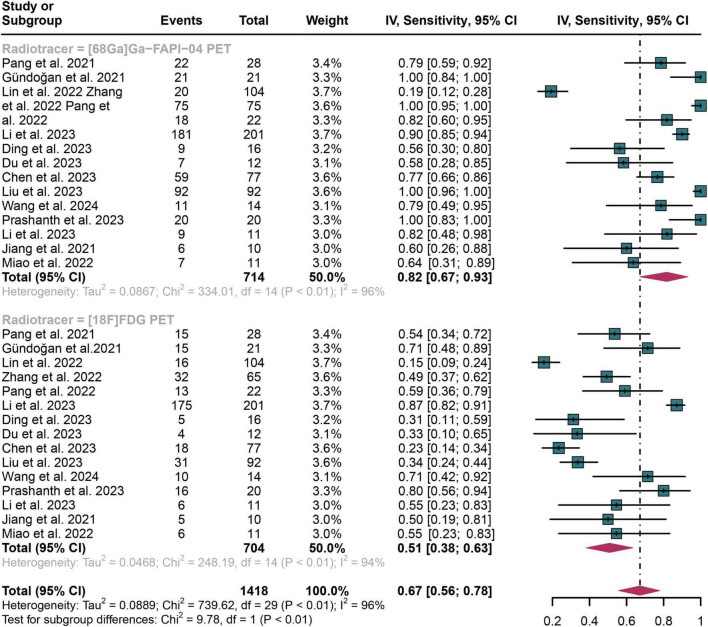
Forest plot shows a sensitivity analysis comparing [^68^Ga]Ga-FAPI-04 PET and [^18^F]FDG PET in detecting lymph node metastasis in digestive system cancers.

For lymph node metastasis diagnosis in digestive system cancers, [^68^Ga]Ga-FAPI-04 PET had a pooled sensitivity of 0.82 (95% CI: 0.67–0.93), while the overall sensitivity of [^18^F]FDG PET was 0.51 (95% CI: 0.38–0.63) (Figure 3). The overall sensitivity of [^68^Ga]Ga-FAPI-04 PET and [^18^F]FDG PET showed statistical difference (*P* < 0.01) (Figure 3).

The I^2^ value was 96% for both [^68^Ga]Ga-FAPI-04 and [^18^F]FDG PET sensitivity. Leave-one-out analyses demonstrated result stability (range: 0.87–0.96 and 0.84–0.95, respectively; Supplementary Figures 1, 2). Meta-regression revealed patient number as a significant source of heterogeneity for [^68^Ga]Ga-FAPI-04 PET (*P* = 0.02), while no covariates significantly influenced [^18^F]FDG PET (Tables 3, 4).

**TABLE 3:** Subgroup analysis and meta-regression analysis for [^68^Ga]Ga-FAPI-04 PET.

Covariate	Studies, n	Sensitivity (95%CI)	*P*-value	Studies, n	Specificity (95%CI)	*P*-value
No. of patients			0.02			0.87
≤50	11	0.88(0.76–0.97)		9	0.88(0.80–0.95)	
>50	4	0.58(0.26–0.87)		3	0.99(0.89–1.00)	
Region			0.15			NA
China	13	0.77(0.61–0.90)		12	0.91(0.84–0.97)	
Non-China	2	1.00(0.95–1.00)		0		
Study design			0.12			0.77
Prospective	7	0.67(0.41–0.89)		6	0.93(0.78–1.00)	
Retrospective	8	0.92(0.80–0.99)		6	0.91(0.82–0.97)	
Reference standard			0.44			0.67
Only pathology	10	0.77(0.55–0.94)		8	0.93(0.83–0.99)	
No-only pathology	5	0.89(0.76–0.98)		4	0.89(0.76–0.97)	
Analysis method			0.79			0.76
Patient based	7	0.80(0.59–0.96)		4	0.90(0.74–1.00)	
Lesion based	8	0.83(0.61–0.97)		8	0.92(0.82–0.98)	
Image modality			0.37			0.55
PET/CT	12	0.64(0.68–0.97)			0.92(0.82–0.98)	
PRT/MRI	2	0.59(0.66–0.80)			0.82(0.57–0.99)	

NA, not available.

**TABLE 4:** Subgroup analysis and meta-regression analysis for [18F]FDG PET.

Covariate	Studies, n	Sensitivity (95%CI)	*P*-value	Studies, n	Specificity (95%CI)	*P*-value
No. of patients			0.73			0.69
≤50	11	0.53(0.39–0.67)		9	0.79(0.56–0.96)	
>50	4	0.45(0.19–0.73)		3	0.86(0.60–1.00)	
Region			0.25			NA
China	13	0.47(0.34–0.60)		12	0.81(0.64–0.94)	
Non-China	2	0.76(0.61–0.88)		0		
Study design			0.88			0.15
Prospective	7	0.45(0.09–0.86)		6	0.91(0.80–0.99)	
Retrospective	8	0.79(0.70–0.86)		6	0.69(0.35–0.95)	
Reference standard			0.93			0.24
Only pathology	10	0.50(0.35–0.64)		8	0.88(0.76–0.96)	
No-only pathology	5	0.52(0.27–0.77)		4	0.66(0.16–1.00)	
Analysis method			0.75			0.33
Patient based	7	0.55(0.40–0.70)		4	0.91(0.78–0.99)	
Lesion based	8	0.48(0.30–0.67)		8	0.76(0.50–0.95)	
Image modality			0.63			0.57
PET/CT	12	0.55(0.41–0.69)		9	0.76(0.41–0.94)	
PRT/MRI	2	0.41(0.20–0.63)		2	0.87(0.73–0.98)	

NA, not available.

### 3.4 Comparing the specificity of [^68^Ga]Ga-FAPI-04 PET and [^18^F]FDG PET in detecting lymph node metastasis of digestive system cancers

**FIGURE 4:**
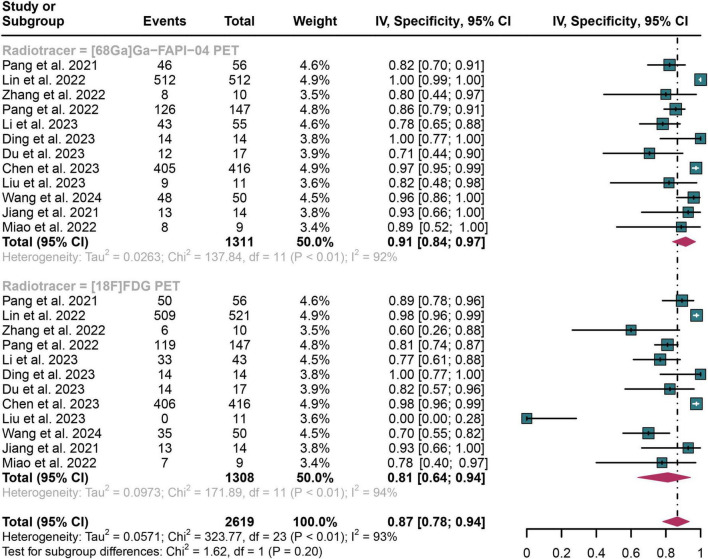
Forest plot shows a specificity analysis comparing [^68^Ga]Ga-FAPI-04 PET and [^18^F]FDG PET in detecting lymph node metastasis in digestive system cancers.

In detecting lymph node metastasis in digestive system cancers, [^68^Ga]Ga-FAPI-04 PET showed an overall specificity of 0.91 (95% CI: 0.84–0.97), compared to a pooled specificity of 0.81 (95% CI: 0.64–0.94) for [^18^F]FDG PET (Figure 4). The total specificity of [^68^Ga]Ga-FAPI-04 PET and [^18^F]FDG PET showed no statistical difference (*P* = 0.20) (Figure 4).

The I^2^ value was 92% for [^68^Ga]Ga-FAPI-04 PET specificity and 94% for [^18^F]FDG PET. Leave-one-out analyses demonstrated result stability (range: 0.83–0.93 and 0.91–0.94, respectively; Supplementary Figures 3, 4). Meta-regression showed no significant impact of study design, patient number, reference standard, or analysis type on either tracer’s specificity (Tables 3, 4).

### 3.5 Comparing the sensitivity and specificity of [^68^Ga]Ga-FAPI-04 PET and [^18^F]FDG PET in detecting lymph node metastasis of specific digestive system cancers

For gastric cancer (for sensitivity, 7 studies with 610 patients were included; for specificity, 6 studies with 1, 965 patients were included), the pooled sensitivity of [^68^Ga]Ga-FAPI-04 PET was 0.74 (95% CI: 0.45–0.95), significantly higher than the 0.40 (95% CI: 0.24–0.57) observed for [^18^F]FDG PET (*P* = 0.04). The specificity of [^68^Ga]Ga-FAPI-04 PET was 0.93 (95% CI: 0.80–1.00), while that of [^18^F]FDG PET was 0.91 (95% CI: 0.77–0.99), showing no significant difference (*P* = 0.73).

For pancreatic cancer (For sensitivity, 2 studies and 76 patients were included; for specificity, 2 studies with 322 patients were included), [^68^Ga]Ga-FAPI-04 PET had a sensitivity of 0.71 (95% CI: 0.43–0.92), compared to 0.46 (95% CI: 0.20–0.73) for [^18^F]FDG PET, with the difference not reaching statistical significance (*P* = 0.21). The specificities were similar, with [^68^Ga]Ga-FAPI-04 PET at 0.93 (95% CI: 0.75–1.00) and [^18^F]FDG PET at 0.92 (95% CI: 0.66–1.00) (*P* = 0.90).

For biliary tract carcinoma (for sensitivity, 1 study and 402 patients were included; for specificity, 1 studies with 98 patients were included), [^68^Ga]Ga-FAPI-04 PET achieved a sensitivity of 0.90 (95% CI: 0.87–0.91), slightly higher than the 0.87 (95% CI: 0.82–0.91) observed with [^18^F]FDG PET, although this difference was not statistically significant (*P* = 0.35). The specificity was 0.78 (95% CI: 0.65–0.88) for [^68^Ga]Ga-FAPI-04 PET and 0.77 (95% CI: 0.61–0.88) for [^18^F]FDG PET, with no significant difference (*P* = 0.86).

For colorectal cancer (for sensitivity, 1 study and 40 patients were included, no data for specificity), only sensitivity data were available. [^68^Ga]Ga-FAPI-04 PET exhibited a sensitivity of 1.00 (95% CI: 0.83–1.00), significantly higher than the 0.80 (95% CI: 0.56–0.94) recorded for [^18^F]FDG PET (*P* = 0.02). Detailed results can be found in Table 5.

**TABLE 5:** Diagnostic performance of [^68^Ga]Ga-FAPI-04 PET vs. [^18^F]FDG PET in lymph node metastasis of specific digestive cancers.

Outcome measure	[^68^Ga]Ga-FAPI-04 PET				[^18^F]FDG PET				*P*-value between [^68^Ga]Ga-FAPI-04 and [^18^F]FDG (*P* < 0.05 was consider significant difference)
	**No. of studies**	**No. of total patients and/or lesions**	**No. of total events**	**Pooled random-effect model results (95% CI)**	**No. of studies**	**No. of total patients and/or lesions**	**No. of total events**	**Pooled random-effect model results (95% CI)**	
Gastric cancer LNM (sensitivity)	7	310	195	0.74(0.45–0.95)	7	300	96	0.40(0.24;0.57)	0.04
Gastric cancer LNM (specificity)	6	978	958	0.93(0.80–1.00)	6	987	955	0.91(0.77–0.99)	0.73
Pancreatic cancer LNM (sensitivity)	2	38	27	0.71(0.43–0.92)	2	38	18	0.46(0.20–0.73)	0.21
Pancreatic cancer LNM (specificity)	2	161	140	0.93(0.75–1.00)	2	161	133	0.92(0.66–1.00)	0.90
Biliary tract carcinoma LNM (sensitivity)	1	201	181	0.90(0.85–0.94)	1	201	175	0.87(0.82–0.91)	0.35
Biliary tract carcinoma LNM (specificity)	1	55	43	0.78(0.65–0.88)	1	43	33	0.77(0.61–0.88)	0.86
Colorectal cancer LNM (sensitivity)	1	20	20	1.00(0.83–1.00)	1	20	16	0.80(0.56–0.94)	0.02

LNM, lymph node metastasis.

### 3.6 Comparing the false positive rates (FPR) and false negative rates (FNR) of [^68^Ga]Ga-FAPI-04 PET and [^18^F]FDG PET in detecting lymph node metastasis of digestive system cancers

**FIGURE 5:**
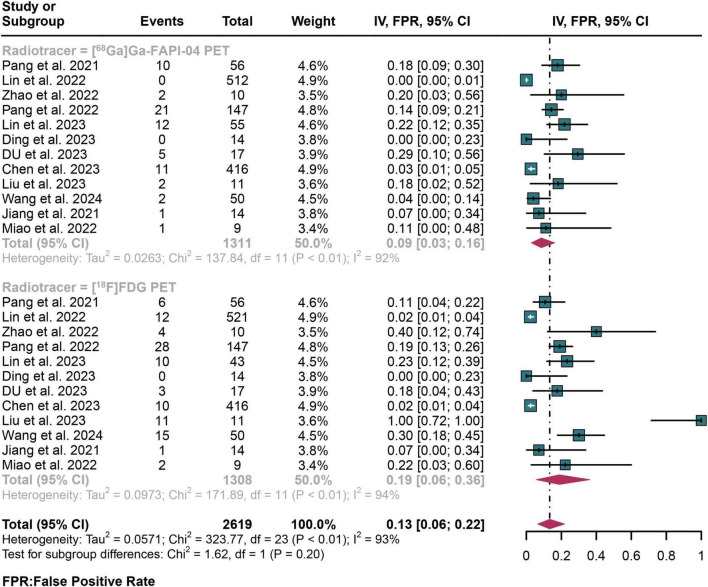
Forest plot shows a false positive rate (FPR) analysis comparing [^68^Ga]Ga-FAPI-04 PET and [^18^F]FDG PET in detecting lymph node metastasis in digestive system cancer.

For lymph node metastasis diagnosis in digestive system cancers, [^68^Ga]Ga-FAPI-04 PET had a pooled FPR of 0.09 (95% CI: 0.03–0.16), while the overall FPR of [^18^F]FDG PET was 0.19 (95% CI: 0.06–0.36) (Figure 5). The overall FPR of [^68^Ga]Ga-FAPI-04 PET and [^18^F]FDG PET showed no statistical difference (*P* = 0.20) (Figure 5).

**FIGURE 6:**
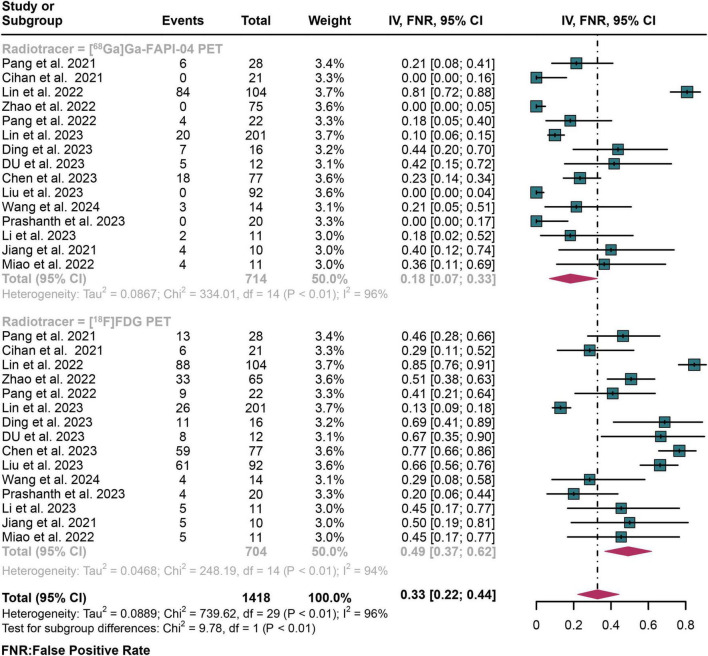
Forest plot shows a false negative rate (FNR) analysis comparing [^68^Ga]Ga-FAPI-04 PET and [^18^F]FDG PET in detecting lymph node metastasis in digestive system cancers.

For lymph node metastasis diagnosis in digestive system cancers, [^68^Ga]Ga-FAPI-04 PET had a pooled FNR of 0.18 (95% CI: 0.07–0.33), while the overall FNR of [^18^F]FDG PET was 0.49 (95% CI: 0.37–0.62) (Figure 6). The overall FNR of [^68^Ga]Ga-FAPI-04 PET and [^18^F]FDG PET showed statistical difference (*P* < 0.01) (Figure 6).

### 3.7 Publication bias

The funnel plot asymmetry test and Egger’s test were conducted to assess potential publication bias in sensitivity estimates. The results indicated no significant publication bias for [^68^Ga]Ga-FAPI-04 PET (Egger’s test: *P* = 0.76) or [^18^F]FDG PET (Egger’s test: *P* = 0.64) (Supplementary Figures 5, 6). In contrast, these tests revealed significant publication bias in the specificity estimates for both [^68^Ga]Ga-FAPI-04 PET (Egger’s test: *P* = 0.02) and [^18^F]FDG PET (Egger’s test: *P* = 0.01, Supplementary Figures 7, 8).

## 4 Discussion

[^18^F]FDG PET is utilized for both the initial staging and subsequent restaging of lymph node metastasis in digestive system cancers, ensuring precise assessment and supporting the optimization of therapeutic strategies (43). Recent studies show that [^68^Ga]Ga-FAPI-04 outperforms [^18^F]FDG PET in diagnosing primary digestive system tumors. However, there is ongoing debate about their relative effectiveness in detecting lymph node metastasis in digestive system cancers. This is the first systematic review and meta-analysis comparing the detection performance of [^68^Ga]Ga-FAPI-04 PET and [18F]FDG PET for identifying lymph node metastasis in digestive system cancers.

A significant difference in sensitivity was found between [^68^Ga]Ga-FAPI-04 PET and [^18^F]FDG PET (*P* < 0.01), although no difference in specificity was observed (*P* = 0.20), consistent with previous studies (37, 39). The diagnostic performance of [^18^F]FDG PET is limited due to the variable physiological uptake of [^18^F]FDG in the gastrointestinal tract, which can interfere with the detection of lesions (44). Additionally, [^18^F]FDG shows reduced uptake in certain histological subtypes, such as adenocarcinoma and signet-ring cell carcinoma, further affecting its diagnostic sensitivity (45, 46). In contrast, FAP, which is highly expressed by cancer-associated fibroblasts (CAFs) and minimally in normal tissues, leads to a lower uptake of [^68^Ga]Ga-FAPI in healthy tissue (47). This results in a higher tumor-to-background ratio (TBR), thereby enhancing tumor visualization, particularly in regions with high glucose metabolism (48, 49). The comparable high specificity of both imaging agents in detecting lymph node metastasis may be due to the fact that both modalities provide functional information. When evaluating specific tumor subgroups-pancreatic cancer (2 studies), cholangiocarcinoma (1 study), and colorectal cancer (1 study)-no significant differences in sensitivity or specificity were found between [^68^Ga]Ga-FAPI-04 PET and [^18^F]FDG PET. However, in a systematic review by Zhuang et al. (included 3 studies), [^68^Ga]Ga-FAPI-04 PET demonstrated a higher positive detection rate for lymph node metastasis in colorectal cancer compared to [^18^F]FDG PET, although sensitivity and specificity were not reported (50). The discrepancies between the findings for specific tumor subgroups and the overall results from the systematic review may be due to the small number of studies and patient samples in our analysis. Additionally, not all lymph node metastases were confirmed by pathological biopsy, with some relying on imaging follow-up as the standard. These factors could contribute to the variability and instability of the results.

Ouyang et al. found that [^68^Ga]Ga-FAPI-04 PET was more effective than [^18^F]FDG PET in diagnosing primary cancers of the digestive system, particularly gastric, liver, biliary tract, and pancreatic cancers, with pooled sensitivity and specificity of 0.98 and 0.81, respectively (20). Multiple studies further confirmed the superior accuracy of [^68^Ga]Ga-FAPI-04 PET in these contexts. However, it is important to note that Ouyang et al. did not assess its efficacy in diagnosing lymph node metastasis. To address this research gap, we conducted the first analysis of [^68^Ga]Ga-FAPI-04 PET for diagnosing lymph node metastasis in digestive system cancers. Our findings revealed that [^68^Ga]Ga-FAPI-04 PET has a higher sensitivity of 0.82, compared to 0.51 for [^18^F]FDG PET. This underscores the superior diagnostic capability of [^68^Ga]Ga-FAPI-04 PET in this specific application.

[^18^F]FDG PET offers advantages such as broad availability, established clinical validation, and valuable metabolic information for tumor assessment. However, it has limitations, including physiological uptake in normal tissues, which complicates lesion detection, and susceptibility to variations in tissue glucose metabolism. In contrast, [^68^Ga]Ga-FAPI-04 PET provides high selectivity for tumor detection, potential for early diagnosis, and theragnostic applications. Our investigation of [^68^Ga]Ga-FAPI-04 PET reveals statistically significant improvements in diagnostic sensitivity (*P* = 0.04) among gastric cancer patients. By precisely targeting fibrotic and tumor microenvironmental markers, this imaging technique demonstrates enhanced staging capabilities. Preliminary findings suggest potential advancements in early diagnostic strategies for specific patient populations. Nonetheless, its limited availability, the need for broader clinical validation, and concerns about radiation exposure are significant drawbacks. The half-life of [^68^Ga]Ga-FAPI-04 is determined by the ^68^Ga radioisotope, with a half-life of 68 min, which poses certain challenges for its preparation and transportation. In contrast, [^18^F]FDG has a longer half-life (109.8 min), potentially offering advantages in transportation and application (51). [^68^Ga]Ga-FAPI-04 synthesis is characterized by extreme sensitivity to reaction conditions, requiring precise technical parameters. The synthesis process demands meticulous control, including temperature regulation precisely at 100°C, specialized reagents like 4-(2-hydroxyethyl) piperazine-1-ethanesulfonic acid, and professional radioisotope synthesis equipment such as cassette-based automated synthesizers (52). Multiple parameters necessitate precise optimization, resulting in a complex synthesis protocol (52). These multifaceted technical complexities may significantly constrain its widespread adoption in clinical practice. The safety assessments in the included studies indicated that no adverse reactions were reported during or after the use of either tracer, suggesting that both are safe for diagnostic purposes. Our findings indicate that [^68^Ga]Ga-FAPI-04 PET offers higher sensitivity and similar specificity compared to [^18^F]FDG PET in detecting lymph node metastasis in digestive system cancers. This improved sensitivity could enhance cancer staging accuracy and reduce the need for additional tests or treatments. FAPI-based radiotracers may significantly advance the diagnosis and treatment of digestive system cancers, particularly gastric cancer, leading to more personalized patient care and improved survival outcomes. Molecular imaging with [^68^Ga]Ga-FAPI-04 demonstrates heightened diagnostic precision in lesion identification, potentially mitigating false-negative occurrences and reducing unnecessary subsequent radiological assessments (53, 54). Notwithstanding these initial promising indicators, rigorous comparative analyses remain imperative to comprehensively evaluate the tracer’s clinical effectiveness and economic rationalization.

Several limitations should be noted. First, study heterogeneity may have affected the sensitivity and specificity results, with meta-regression suggesting that sample size (>50, <50) could be a contributing factor. Second, our results indicate significant publication bias in the specificity estimates for both [^68^Ga]Ga-FAPI-04 PET and [^18^F]FDG PET. This bias may primarily stem from the substantial overrepresentation of Chinese populations in the literature, with the majority of included studies originating from Chinese research centers. Third, due to technical and ethical constraints, not all positive lesions were confirmed by histopathology, necessitating the use of morphological criteria and follow-up imaging as reference standards. Fourth, the limited number of head-to-head studies has hindered our ability to compare the diagnostic performance of tools for specific gastrointestinal tumors, such as liver, duodenal, and appendiceal cancers. To address this gap, future research should prioritize head-to-head studies on these specific tumors. Additionally, to validate the current findings, further research involving diverse populations and well-designed prospective studies is required.

## 5 Conclusion

[^68^Ga]Ga-FAPI-04 PET showed higher sensitivity but comparable specificity to [^18^F]FDG PET in detecting lymph node metastasis of digestive system cancers, particularly in gastric cancer. Both tracers performed similarly in biliary tract, pancreatic, and colorectal cancers. However, due to the limited sample sizes and notable heterogeneity observed in the current studies, there is a pressing need for larger prospective multicenter studies. Such research should focus on underrepresented populations outside of China and specific cancer subtypes, such as liver and duodenal cancers, to enhance the generalizability of findings and validate the efficacy of [^68^Ga]Ga-FAPI-04 PET in diverse clinical settings.

## Key points

Question: Is [^68^Ga]Ga-FAPI-04 PET more effective than [^18^F]FDG PET in detecting lymph node metastasis in patients with digestive system cancers?

Pertinent findings: In a meta-analysis of 15 studies including 617 patients, [^68^Ga]Ga-FAPI-04 PET showed significantly higher sensitivity (0.82 vs. 0.51) and similar specificity (0.91 vs. 0.81) compared to [^18^F]FDG PET for detecting lymph node metastasis in digestive system cancers.

Implications for patient care: [^68^Ga]Ga-FAPI-04 PET may provide more accurate lymph node staging in patients with digestive system cancers, potentially leading to better treatment planning and patient outcomes.

## Data Availability

The original contributions presented in the study are included in the article/Supplementary material, further inquiries can be directed to the corresponding author.
