# Supplementary material for: Diagnostic performance of [68Ga]Ga-FAPI-04 PET vs. [18F]FDG PET in detecting lymph node metastasis in digestive system cancers: a head-to-head comparative meta-analysis
Source: Front Med (Lausanne). 2025 Mar 21;12:1541461. doi: 10.3389/fmed.2025.1541461 (PMC11968752; doi:10.3389/fmed.2025.1541461)

Supplementary Table 1 Search strategy in PubMed, Embase and Web of Science.

| Database | Search strategy |
| --- | --- |
| PubMed | ("Positron-Emission Tomography"[Mesh] OR "PET" [Title/Abstract] OR "Positron Emission Tomography" [Title/Abstract] OR "Positron-Emission Tomography Imaging" [Title/Abstract]) AND ("68Ga-FAPI"[Title/Abstract] OR "FAPI-04"[Title/Abstract] OR "FAPI"[Title/Abstract] OR "fibroblast activation protein"[Title/Abstract] OR "FAP"[Title/Abstract]) AND ("Digestive System Neoplasms"[Mesh] OR “Digestive”[Title/Abstract] OR “Gastric”[Title/Abstract] OR “Gastrointestinal”[Title/Abstract] OR “Pancreatic”[Title/Abstract] OR “Pancreas”[Title/Abstract] OR “Pancreatic”[Title/Abstract] OR “Colorectal”[Title/Abstract] OR “Hepatic”[Title/Abstract] OR “Hepatocellular”[Title/Abstract] OR “Liver”[Title/Abstract] OR “Cholangiocarcinoma”[Title/Abstract]) |
| Embase | ('positron emission tomography'/exp OR ‘PET’:ab,ti OR ‘Positron Emission Tomography’:ab,ti OR ‘Positron-Emission Tomography Imaging’:ab,ti) AND ('fapi 04 ga 68'/exp OR ‘68Ga-FAPI’:ab,ti OR ‘FAPI-04’:ab,ti OR ‘FAPI’:ab,ti OR ‘fibroblast activation protein’:ab,ti OR ‘FAP’:ab,ti) AND ('digestive system tumor'/exp OR ‘Digestive’:ab,ti OR ‘Gastric’:ab,ti OR ‘Gastrointestinal’:ab,ti OR ‘Pancreatic’:ab,ti OR ‘Pancreas’:ab,ti OR ‘Pancreatic’:ab,ti OR ‘Colorectal’:ab,ti OR ‘Hepatic’:ab,ti OR ‘Hepatocellular’:ab,ti OR ‘Liver’:ab,ti OR ‘Cholangiocarcinoma’:ab,ti) AND ('sensitivity and specificity'/exp OR ‘sensitivity’:ab,ti OR ‘specificity’:ab,ti) |
| Web of Science | ((TS=("Positron-Emission Tomography" OR "PET" OR "Positron Emission Tomography" OR "Positron-Emission Tomography Imaging")) AND TS=("68Ga-FAPI" OR "FAPI-04" OR "FAPI" OR "fibroblast activation protein" OR "FAP")) AND TS=("Digestive System Neoplasms" OR “Digestive” OR “Gastric” OR “Gastrointestinal” OR “Pancreatic” OR “Pancreas” OR “Pancreatic” OR “Colorectal” OR “Hepatic” OR “Hepatocellular” OR “Liver” OR “Cholangiocarcinoma”) |

Supplementary Figure 1 The leave-one-out sensitivity analysis of [^68^Ga]Ga-FAPI-04 PET’s ability to detect lymph node metastasis in digestive system cancers.


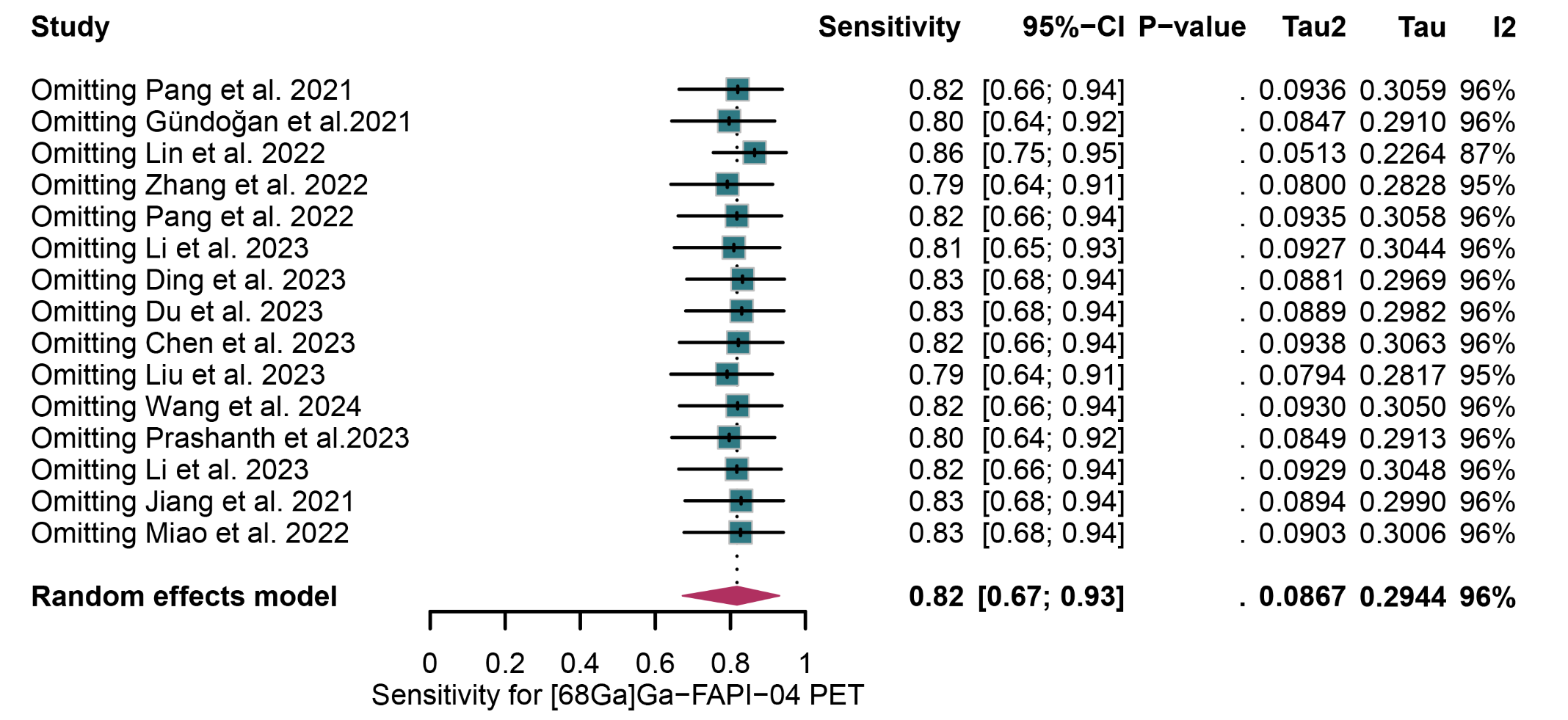


Supplementary Figure 2: The leave-one-out sensitivity analysis of [^18^F]FDG PET’s ability to detect lymph node metastasis in digestive system cancers.


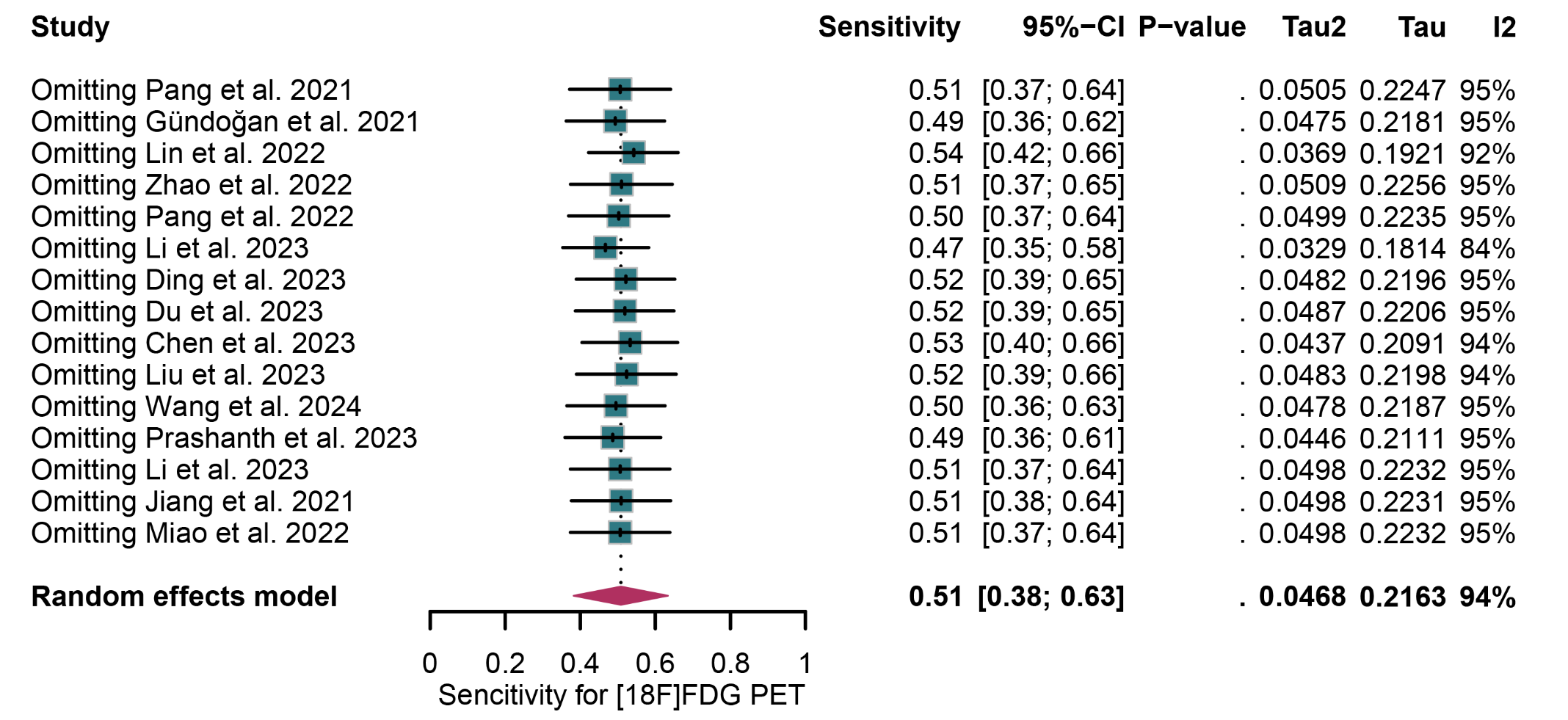


Supplementary Figure 3: The leave-one-out specificity analysis of [^68^Ga]Ga-FAPI-04 PET’s ability to detect lymph node metastasis in digestive system cancers.


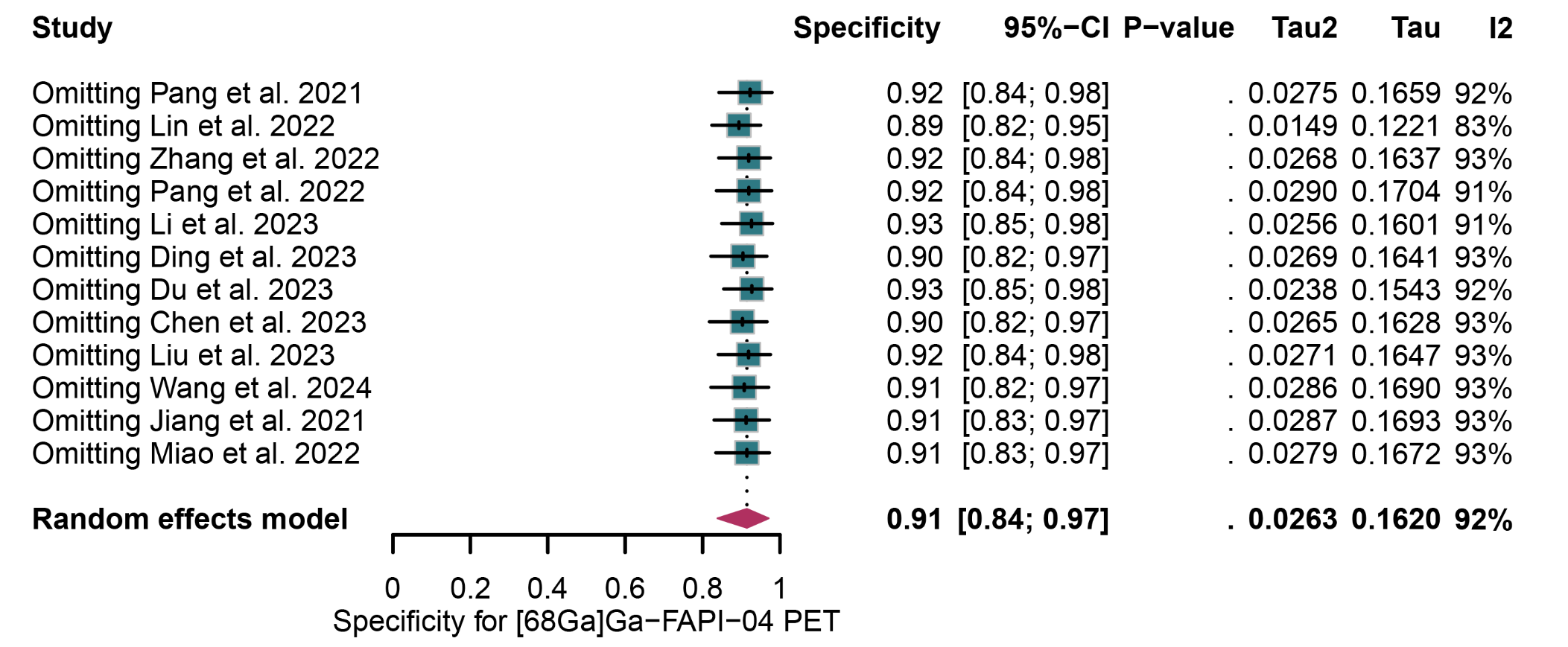


Supplementary Figure 4: The leave-one-out specificity analysis of [^18^F]FDG PET’s ability to detect lymph node metastasis in digestive system cancers.


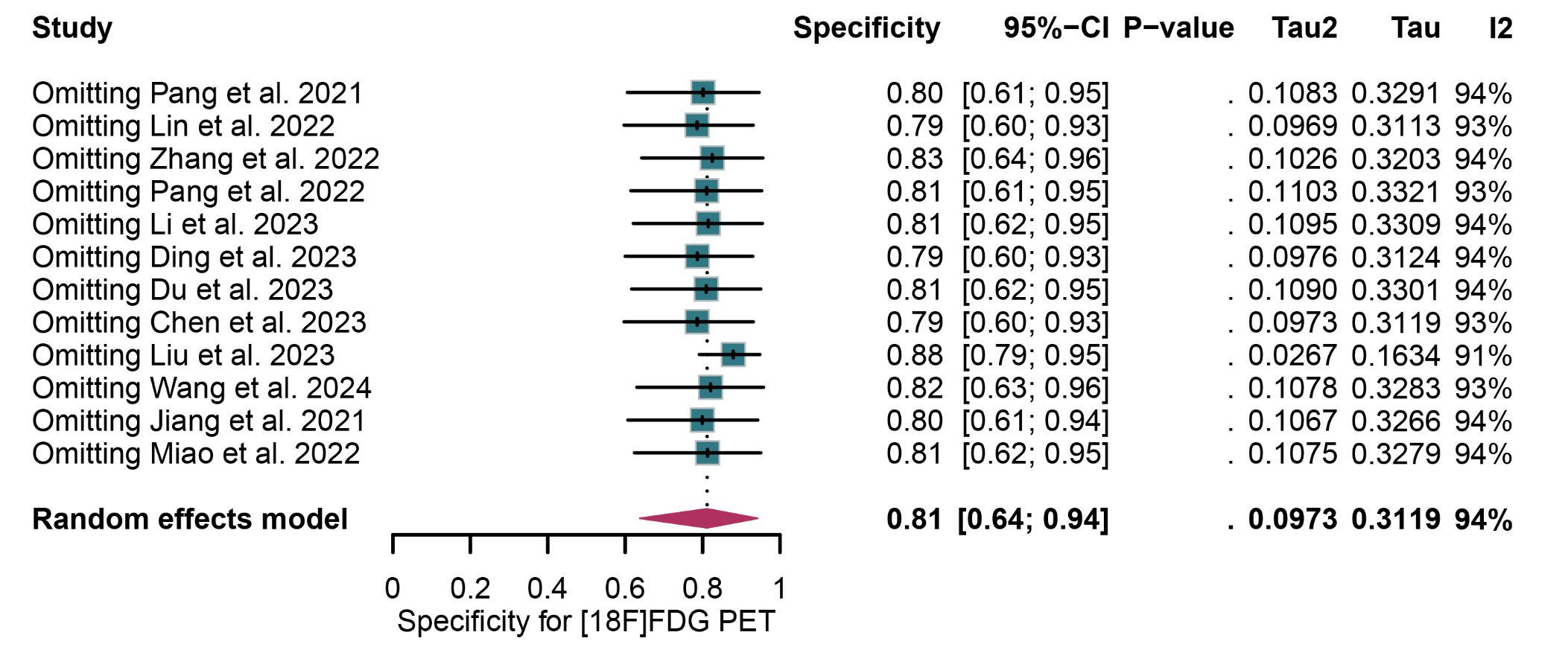


Supplementary Figure 5: The Deeks' funnel plot of sensitivity of [^68^Ga]Ga-FAPI-04 PET.( *P*＜0.05 was considered significant.)


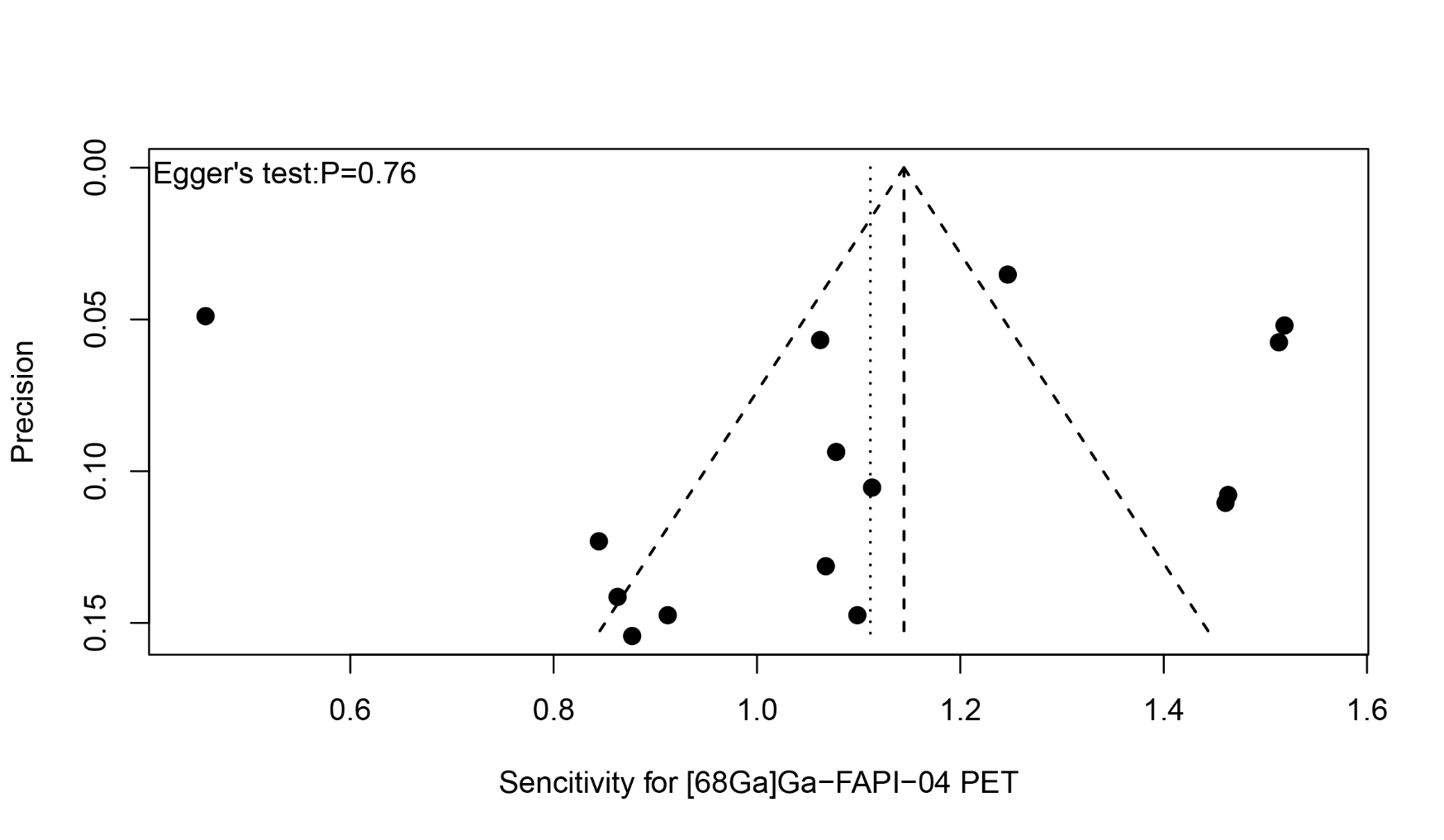


Supplementary Figure 6: The Deeks' funnel plot of sensitivity of [^18^F]FDG PET.( *P*＜0.05 was considered significant.)


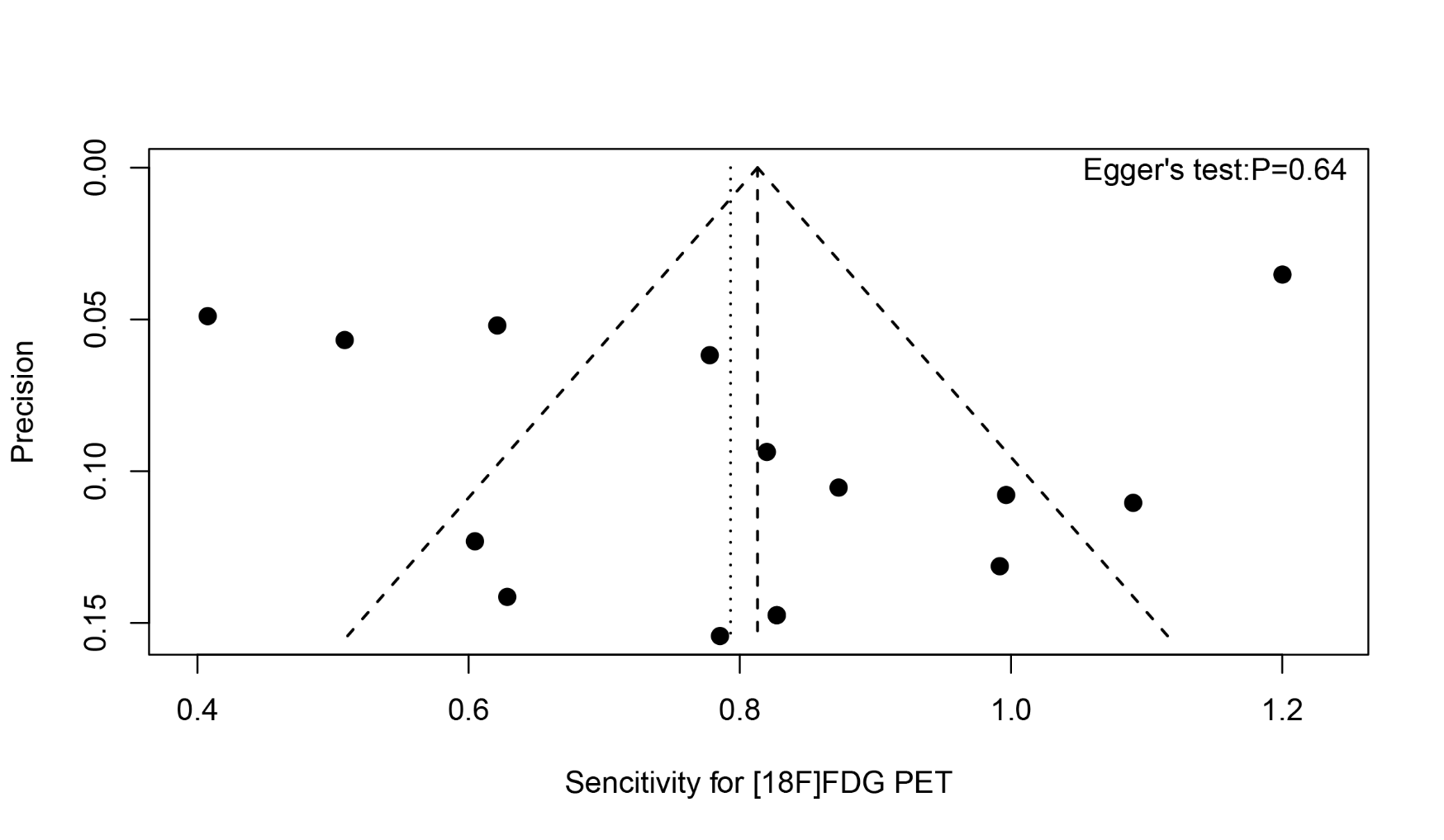


Supplementary Figure 7: The Deeks' funnel plot of specificity of [^68^Ga]Ga-FAPI-04 PET.( *P*＜0.05 was considered significant.)


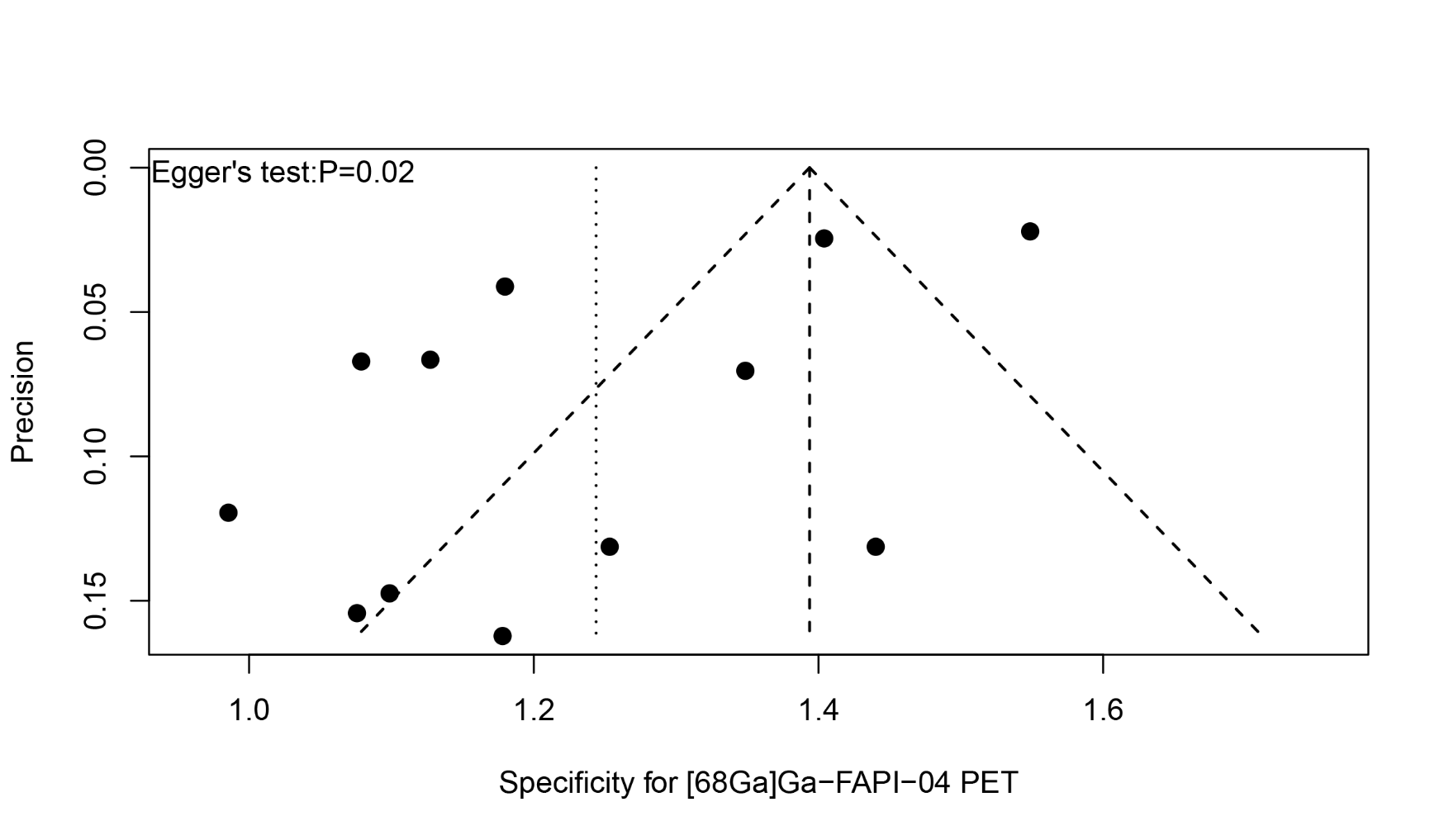


Supplementary Figure 8: The Deeks' funnel plot of specificity of [^18^F]FDG PET.( *P*＜0.05 was considered significant.)


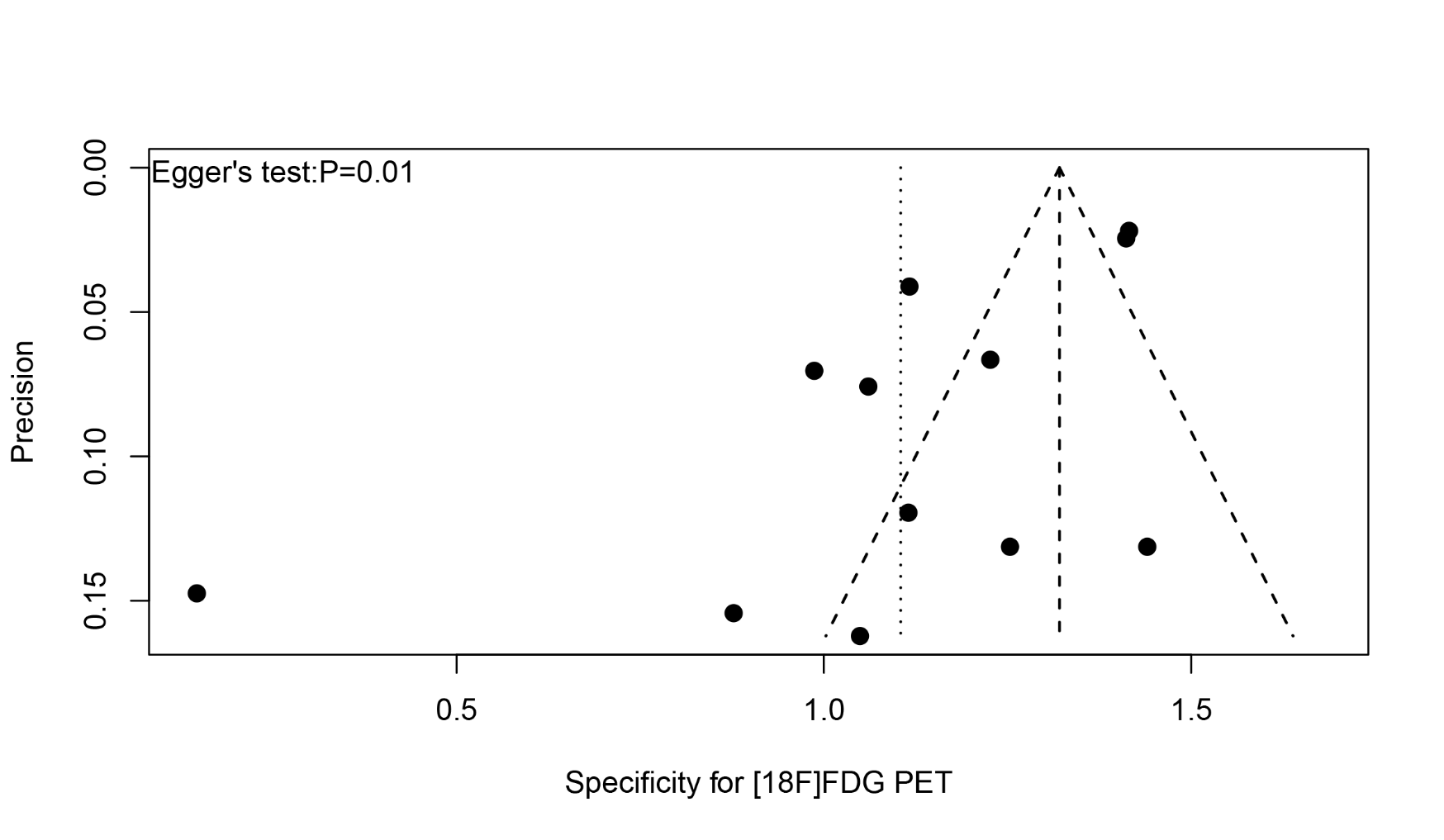

Supplement: Supplementary file 1 [file Data_Sheet_1.docx]
